# A Pseudo-Labeling Multi-Screening-Based Semi-Supervised Learning Method for Few-Shot Fault Diagnosis

**DOI:** 10.3390/s24216907

**Published:** 2024-10-28

**Authors:** Shiya Liu, Zheshuai Zhu, Zibin Chen, Jun He, Xingda Chen, Zhiwen Chen

**Affiliations:** 1 College of Mechanical Engineering and Automation, Foshan University, Foshan 528200, China; liushiya@fosu.edu.cn (S.L.); 2112052078@stu.fosu.edu.cn (Z.Z.); 2112202070@stu.fosu.edu.cn (Z.C.); 2112202075@stu.fosu.edu.cn (X.C.); 2 School of Automation, Central South University, Changsha 410083, China; zhiwen.chen@csu.edu.cn

**Keywords:** few-shot learning, pseudo-labeling, prototypical network, AdaBoost adaptation

## Abstract

In few-shot fault diagnosis tasks in which the effective label samples are scarce, the existing semi-supervised learning (SSL)-based methods have obtained impressive results. However, in industry, some low-quality label samples are hidden in the collected dataset, which can cause a serious shift in model training and lead to the performance of SSL-based method degradation. To address this issue, the latest prototypical network-based SSL techniques are studied. However, most prototypical network-based scenarios consider that each sample has the same contribution to the class prototype, which ignores the impact of individual differences. This article proposes a new SSL method based on pseudo-labeling multi-screening for few-shot bearing fault diagnosis. In the proposed work, a pseudo-labeling multi-screening strategy is explored to accurately screen the pseudo-labeling for improving the generalization ability of the prototypical network. In addition, the AdaBoost adaptation-based weighted technique is employed to obtain accurate class prototypes by clustering multiple samples, improving the performance that deteriorated by low-quality samples. Specifically, the squeeze and excitation block technique is used to enhance the useful feature information and suppress non-useful feature information for extracting accuracy features. Finally, three well-known bearing datasets are selected to verify the effectiveness of the proposed method. The experiments illustrated that our method can receive better performance than that of the state-of-the-art methods.

## 1. Introduction

As an indispensable component in industrial applications, mechanical equipment is an important force in promoting sustainable development and industrial upgrading [1,2]. However, any tiny failure may cause production downtime or even catastrophic consequences. Furthermore, it is prone to component failure when the mechanical equipment operates under high loads for a long time [3]. It is of great significance to carry out an equipment fault diagnosis study to improve equipment safety and reliability, which has attracted increasing attention in the industrial safety community [4,5].

In the last decade, there has been a rapid development of information technology, which brings new perspectives and challenges for the traditional fault diagnosis methods of rotating machinery and promotes the development of fault diagnosis from traditional shallow models to deep learning models. Zhang et al. [6] proposed an improved residual network (ResNet) based on hybrid attention for wind turbine gearbox fault diagnosis. Shao et al. [7] presented a novel deep belief network (DBN) based on convolutional for bearing fault diagnosis. He et al. [8] explored a transfer learning fault diagnosis method based on a convolutional neural network (CNN), Shao et al. [9] provided a modified stacked autoencoder (SAE) based on adaptive Morlet wavelet for rotating machinery fault diagnosis. Nie et al. [10] developed a fault diagnosis framework to relax the impact of noisy labels with recurrent neural networks (RNN). These existing deep learning models can achieve results and overcome the shortcomings of shallow models, which heavily rely on manual feature extraction. However, the number of samples selected to train the deep model will seriously affect the deep model training accuracy. Moreover, in real industrial applications, it is difficult or even impossible to collect a large amount of label data, which gives the deep learning-based methods poor generalization ability. To this end, it is essential to capture discriminative knowledge from limited training data to obtain a generalized deep model.

FSL (FSL) is an impressive scenario to utilize limited labeled samples to quickly learn and achieve stable classification results, which has received widespread attention and obtained encouraging progress [11,12]. Up to now, there several FLS methods have been reported, such as Prototypical Network (ProNet) [13,14], Match Network (MatNet) [15,16], Siamese Network (SiaNet) [17,18], etc. Among them, the ProNet transforms the classification problem into a distance measurement problem in the feature embedding space, which has lower time complexity and wildly applied in pattern recognition fields. Chowdhury et al. [19] used the maximum mean discrepancy (MMD) to evaluate the influence of distributions, including and excluding the sample and obtained the sample weights by subtracting from 1 only. Wang et al. [20] presented a weight prototypical network for bearing fault diagnosis, and the Kullback–Leibler (KL) divergence was adopted to estimate the influence of specific samples from a sample distribution. Gao et al. [21] designed a novel prototypical network for noisy few-shot problems based on instance-level and feature-level attention schemes to accentuate the significance of instances and features, respectively. Ye et al. [22] proposed a learning with a strong teacher framework for few-shot learning, in which a strong classifier was constructed to supervise the few-shot learner for image recognition. Zhao et al. [23] employed a dual adaptive representation alignment network for cross-domain few-shot learning, which can update the support instances as prototypes and renew the prototypes with the differentiable. To this end, the above-mentioned FSL-based methods provide a new idea and make certain progress in solving the problem of training scarce samples. However, they only focus on how to evaluate the weight of samples and do not overcome the limitation of small samples. To tackle the problem of FSL from the root, semi-supervised learning (SSL), utilizing a few labeled samples and massive unlabeled samples to improve learning performance, can be divided into three categories: adversarial generation, consistency regularization, and pseudo-labeling. Pseudo-labeling techniques—explored to label unlabeled samples, which are easier to obtain for expanding the training set—have been paid increasing attention recently. He et al. [24] proposed a semi-supervised prototypical network based on pseudo-labeling for bearing fault diagnosis. A fixed threshold was used to select pseudo-labeling and obtain the optimal threshold by a large number of experiments. Fan et al. [25] presented a semi-supervised fault diagnosis method, screened pseudo-labeling with thresholds, and adjusted the dependence of the model on pseudo-labeling through learning. Zhang et al. [26] explored a self-training semi-supervised method that selects unlabeled data with high predictive confidence on a trained model and extracts pseudo-labeling iteratively. Zhang et al. [27] adopted Monte Carlo uncertainty as the threshold to screen the pseudo-labeling and built a gearbox fault diagnosis scenario with small samples based on a momentum prototypical network. Zhou et al. [28] designed an adaptive prototypical network for few-shot learning with sample quality and pseudo-labeling screening to weaken the impact of unreliable pseudo-labeling.

Most of these existing SSL methods based on pseudo-labeling learning achieved impressive accuracy in few-shot learning tasks by increasing the number of training samples by labeling the unlabeled samples. However, there are still the following limitations: (1) A single threshold selected for pseudo-labeling screening cannot guarantee the accuracy of pseudo-labeling collections, which dramatically degrades the performance of SSL methods. (2) Insufficient consideration of iteration-stopping conditions can easily lead to the propagation and accumulation of incorrect information during the iteration process. To resolve the trouble of network degradation caused by the low accuracy of pseudo-labeling screening and insufficient selection samples, a semi-supervised learning method based on a pseudo-labeling multi-screening strategy for a few-shot bearing fault diagnosis is proposed. In this paper, a composite threshold for pseudo-labeling screening combined with Monte Carlo uncertainty and classification probability is explored to overcome the limitations of pseudo-labeling screening with a single threshold. Then, a multi-pseudo-labeling accumulation model based on network optimization is employed to solve the problem of network degradation caused by mislabeling. Finally, three well-known bearing datasets were used to verify the effectiveness of the proposed model. The main contributions are summarized as follows:

(1) A multi-screening strategy based on Monte Carlo uncertainty and classification was proposed for pseudo-labeling selection, which can assist in ensuring the accuracy of pseudo-labeling screening and improve generalization ability.

(2) A semi-supervised learning method based on AdaBoost adaptation was explored to integrate multiple samples into a class prototype to obtain a more accurate class prototype, which can overcome the drawbacks caused by low-quality label samples hidden in the dataset.

(3) An estimation strategy for individual sample contribution rate was presented to accurately obtain individual sample weights for improving the performance of AdaBoost adaptation, which can tackle the problem that ignored the impact from individual differences.

## 2. Theoretical Background

### 2.1. Few-Shot Learning

Learning classification information from limited labeled samples to new samples to improve generalization ability, FSL increases attention in pattern recognition and has achieved interesting progress [29,30].

For a typical FSL task *T*, the whole dataset was described as $S_{T}=\left(S_{trn},S_{tst}\right)$, where the $S_{trn}={\left\{\left(x_{i},y_{i}\right)\right\}}_{i=1}^{N_{trn}}$ and $S_{tst}=\left\{x_{j}\right\}$ denoted the training set and testing set with $x_{i},x_{j}\in X_{T}\subset X$, $y_{i}\in Y_{T}\subset Y$, respectively. The samples $x_{i},x_{j}$ come from a particular domain $D_{T}=\left\{X_{T},P\left(X_{T}\right)\right\}$, which consists of a data space $X_{T}$ and a marginal probability distribution $P(X_{T}).$ In task *T*, the *K* samples were selected from *N* and randomly classed in $S_{trn}$, i.e., *N*-way, *K*-shot [31], aiming to generate an objective prediction function f∈F:X→Y to predict the samples in $S_{tst}.$ It was an enormous challenge to achieve a high-accuracy model in the training process under the limited samples in $S_{trn}.$ Consequently, in the majority of instances, a supervised query dataset is employed: $S_{Q}={\left(x_{i}^{q},y_{i}^{q}\right)}_{i=1}^{N_{sup}},x_{i}^{q}\in X_{\varrho }\subset X,y_{i}^{q}\in Y_{\varrho }\subset Y$, which was selected to randomly assess task *T*.

### 2.2. Prototypical Network

As one of the most attention-attracting techniques of FSL, the prototypical network (PN) aims to generate a prototype for each class with labeled data being widely applied to image recognition and fault diagnosis [32,33].

Specifically, given a support set $S_{s}$, the average of the feature vectors from the same class was used to define the prototype; thus, prototype $P_{l}$ of class *l* is expressed as:

(1) $$P_{l}=\frac{1}{N}\sum_{x_{s}^{l}\in S_{s}}f_{\varphi }\left(x_{n}^{l}\right)$$

where $f_{\varphi }\left(\cdot \right)$ is the feature extractor, $x_{n}^{l}$ represents the *n*-th sample of class *l*, and *N* denotes the number of samples in class *l*.

For unlabeled samples, the Euclidean distance between the sample and the prototypes of each category is calculated, which is normalized by the Softmax function to obtain the probability that the sample belongs to each class. The metric-based meta-learning method of prototypical networks is an effective means of alleviating the overfitting problem that can arise when insufficient data are available.

### 2.3. AdaBoost

Ensemble learning is a type of method that combines multiple models through specific mechanisms to obtain a more robust model. As a classic ensemble algorithm, the main idea of AdaBoost is to construct a strong classifier by the linear combination of several weak classifiers [34]. The performance requirements for weak classifiers need not be too high, they just need to be better than random assumptions. Weak classifiers with high accuracy were given higher weights; conversely, their weights were reduced. Assuming the error rate of a weak classifier is ρ, its weight is:

(2) $$\partial =\frac{1}{2}\log\left(\frac{1-\rho }{\rho }\right)$$

Thus, multiple base classifiers are weighted and combined to improve classification performance:

(3) $$G\left(x\right)=sign\left(\sum \partial H(x)\right)$$

## 3. Semi-Supervised Learning Method of the Proposed

**Figure 1:**
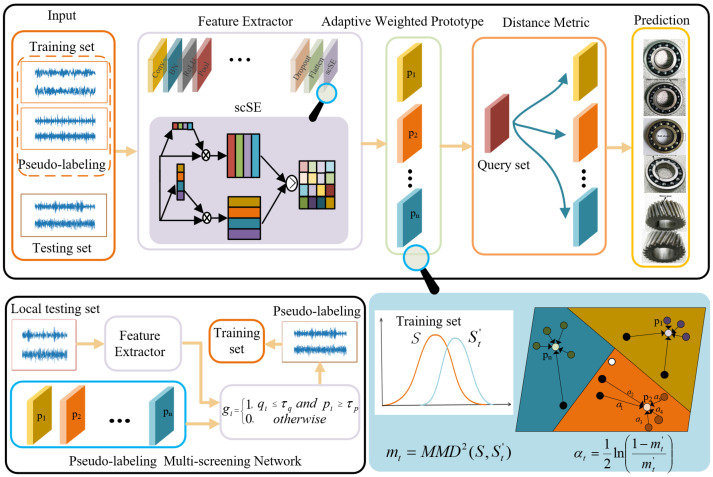
The overall structure diagram of the proposed method.

In this section, a pseudo-labeling multi-screening-based semi-supervised learning method for few-shot fault diagnosis is proposed. The overall structure is presented in Figure 1, which includes three main components: (1) AdaBoost-based adaptive weighted prototypical network (AWPN); (2) pseudo-labeling multi-screening strategy; and (3) semi-supervised learning-based fault diagnosis.

### 3.1. Squeeze and Excitation-Based Feature Extractor

To make the model pay attention to the differences between different perspectives in the learning process and automatically learn the importance of features from different perspectives, Roy et al. [35] proposed spatial and channel squeeze and channel excitation (scSE) for achieving feature recalibration in both space and channel.

**Figure 2:**
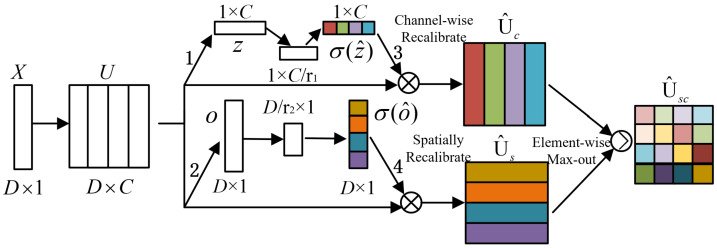
Architectural configuration of scSE.

The application of scSE to one-dimensional data is given in Figure 2. Given that an input feature set $U=\left[u_{1},u_{2},\cdots ,u_{c}\right]\in R^{D\times C}$ is a combination of *C* channels $u_{i}\in R^{D\times 1}$, and can also be rewritten as a combination of *D* feature layer slices $U=[u^{1},u^{2},...,u^{D}]$. Vector $z\in R^{1\times C}$ is generated by spatial squeeze, which is executed by the global average pooling layer, and vector $o\in R^{D\times 1}$ is generated by channel squeeze, obtained by convolution $W_{s}*U$, $W_{s}\in R^{1\times C\times l}$. It is actually a projection of multi-channel features at a feature level. The vector *z* is converted into vector $\hat{z}$ through two fully connected layers $W_{1}^{C}\in R^{C\times \frac{C}{r_{1}}},W_{2}^{C}{\in \;}^{C\times \frac{C}{r_{1}}}$, where $r_{1}$ represents the bottleneck in the channel excitation. To ensure that the excitation channel remains within an appropriate range, $\hat{z}$ is mapped to [0, 1] through a sigmoid function $\sigma (\hat{z}).$ It is worth noting that after channel squeeze, the obtained feature projection is still applicable to the encode–decode operation. Therefore, two fully connected layers $W_{1}^{S}\in R^{D\times \frac{D}{r_{2}}},W_{2}^{S}\in R^{\frac{D}{r_{2}}\times D}$ are used to convert *o* to $\hat{o}$. $r_{2}$ represents the bottleneck in the spatial excitation, and the sigmoid function $\sigma (\hat{o})$ is also used to keep $\hat{o}$ within an appropriate range. Finally, the calibrated features are used in a max-out manner.

The calculation process of scSE is shown in Equations (4) to (10):

(4) $$z=\frac{1}{D}\sum_{i=1}^{D}u\left(i\right)$$

(5) $$\hat{z}=W_{1}^{c}\left(\delta \left(W_{2}^{c}z\right)\right)$$

(6) $${\hat{U}}_{c}=\left[\sigma \left({\hat{z}}_{1}\right)u_{1},\sigma \left({\hat{z}}_{2}\right)u_{2},\cdots ,\sigma \left({\hat{z}}_{C}\right)u_{C}\right]$$

(7) $$o=W_{s}*U$$

(8) $$\hat{o}=W_{1}^{s}\left(\delta \left(W_{2}^{s}o\right)\right)$$

(9) $${\hat{U}}_{s}=\left[\sigma \left({\hat{o}}_{1}\right)u^{1},\sigma \left({\hat{o}}_{2}\right)u^{2},\cdots ,\sigma \left({\hat{o}}_{D}\right)u^{D}\right]$$

(10) $${\hat{U}}_{sc}=\max\left({\hat{U}}_{c},{\hat{U}}_{s}\right)$$

where δ(·) and σ(·) are the ReLU function and sigmoid function, respectively. * represents the convolution operation.

### 3.2. Adaptive Weighted Prototypical Network

Inspired by the AdaBoost theory that weak classifiers can be integrated into strong classifiers, a prototypical network is proposed that adaptively weights the features into strong feature representation. First, each sample is treated as a weak classifier, its weight is calculated by measuring the influence of missing specific samples against the whole sample distribution, which is weighted to build a strong feature representation, that is, class prototype.

As a commonly used criterion, maximum mean discrepancy is adopted to widely measure the distribution discrepancy between the two domains. For a given feature set $U=\left\{f_{\varphi }\left(x_{1}\right),f_{\varphi }\left(x_{2}\right),\ldots ,f_{\varphi }\left(x_{n}\right)\right\},f_{\varphi }\left(\cdot \right)$ is the feature extractor based on squeeze and excitation mechanism. $U_{t}^{\prime }=\left\{f_{\varphi }\left(x_{1}\right),f_{\varphi }\left(x_{2}\right),\ldots f_{\varphi }\left(x_{t-1}\right),f_{\varphi }\left(x_{t+1}\right),\ldots f_{\varphi }\left(x_{n}\right)\right\}$ represents the absence of feature $f_{\varphi }\left(x_{t}\right)$ in feature set *U*. Therefore, the influence of sample $x_{t}$ against the distribution of the sample set can be converted to calculate the maximum mean distribution discrepancy between *U* and $U_{t}^{\prime }$, as shown below:

(11) $$\begin{array}{cc} m_{t} & =MMD^{2}\left(U,U_{t}^{\prime }\right)\\ \; & ={∥\frac{1}{n}\sum_{i=1}^{n}\phi \left(f_{\varphi }\left(x_{i}\right)\right)-\frac{1}{n-1}\sum_{i=1,i\neq t}^{n}\phi \left(f_{\varphi }\left(x_{i}\right)\right)∥}_{H}^{2} \end{array}$$

where ϕ(·) represents the mapping function ${∥x∥}_{H}={\sqrt{<x,x>}}_{H}$ for any x∈H. The smaller $m_{t}$ is, the closer the samples are, and vice versa; the sample deviates from the sample distribution. When $m_{t}=0,U$ and $U_{t}^{\prime }$ are the same distribution. AdaBoost does not have high performance requirements for weak classifiers and only needs to be better than the random hypothesis, so $m_{t}$ is projected to [0, 0.5) by (12).

(12) $$m_{t}^{\prime }=\frac{1}{1+e^{m_{t}}}-0.5$$

Therefore, the weight of feature extractor $f_{\varphi }\left(x_{t}\right)$ is rewritten:

(13) $$\alpha_{t}=\frac{1}{2}\ln\left(\frac{1-m_{t}^{\prime }}{m_{t}^{\prime }}\right)$$

Noting that $m_{t}^{\prime }$ may be set to 0, a sufficiently small positive number ε should be added into the denominator term in (13).

Define the support set $S_{s}=\left\{\left(x_{1},y_{1}\right),\left(x_{2},y_{2}\right),\ldots ,\left(x_{n},y_{n}\right)\right\},y_{i}\in \left\{1,2,\ldots L\right\}$ as the label for L-class samples. $S_{l}$ is the set of samples with the labeled class *l*, and the prototype $P_{l}$ of support class *l* can be calculated by

(14) $$P_{i}=\frac{\sum_{i=1}^{n}\left(x_{i},y_{i}\right)\in S_{i}\alpha_{i}f_{\varphi }\left(x_{i}\right)}{\sum_{i=1}^{n}\alpha_{i}}$$

Assuming that sample *x* needs to be classified, the feature extractor is used to obtain the feature space, and then the Euclidean distance d(·) is adopted to compute the $f_{\phi }\left(x\right)$ and L prototype vectors, respectively. The probability that sample *x* belongs to category *l* is:

(15) $$\mathrm{Pr}\left(y=l|x\right)=\frac{\exp(-d\left(f_{\phi }\left(x\right),P_{i}\right))}{\sum_{i=1}^{L}\exp\left(-d\left(f_{\phi }\left(x\right),P_{i}\right)\right)}$$

Therefore, the loss function J(x,y) of the query set $S_{q}$ is:

(16) $$J\left(x,y\right)=\sum_{\left(x,y\right)\in S_{q}}\log p\left(y=l|x\right)$$

### 3.3. Pseudo-Labeling Multi-Screening Strategy

For data-driven classification models, the number of trainable samples affects the accuracy of the model, especially in semi-supervised learning. In this paper, a pseudo-labeling multi-screening strategy based on uncertainty and classification probability is proposed, which can effectively expand the training set and improve the model training accuracy.

There is a positive correlation between network correction and model output uncertainty. The lower the uncertainty of the model, the smaller the network correction error, and the higher the accuracy of the model [36]. Therefore, the uncertainty of the model is used as one of the indicators of pseudo-label screening. The uncertainty of the output of each sample is calculated by using the Monte Carlo dropout model. In the forward propagation of dropout layers in the testing phase, the Monte Carlo dropout was employed to generate output distributions that emulate the variability observed across different network architectures. The predictive outcomes and the uncertainty of the model are calculated by averaging the outputs and the statistical variance.

Supposing $f_{dt}^{PN}=\left\{f_{d0}^{PN}\left(x\right),\ldots ,f_{dt}^{PN}\left(x\right)\right\}$ is the output of PN after t iterations of random dropout, its uncertainty can be calculated as in Equation (17) [37].

(17) $$\;q=\frac{1}{t}\sum_{j=0}^{t}{\left[f_{dj}^{PN}\left(x\right)-\mu \right]}^{2}$$

where $\mu =\frac{1}{t}\sum_{j=0}^{t}f_{dj}^{PN}\left(x\right)$ represents the predicted posterior mean. The model architectures do not need to be modified, which can reduce the overfitting of the network and improve the computation efficiency. It assesses the predictive mean and model uncertainty by collecting the results of stochastic forward passes.

A pseudo-labeling multi-screening strategy based on the dual threshold of Monte Carlo dropout uncertainty and softmax output probability is constructed as:

(18) $$g_{i}=\left\{\begin{array}{cc} 1, & q_{i}\leq \tau_{q}\;and\;p_{i}\geq \tau_{p}\\ 0, & \;otherwise \end{array}\right.$$

where $q_{i}$ is the estimated uncertainty of sample $i,p_{i}$ is the maximum value of the predicted probability. $\tau_{p}$ and $\tau_{q}$ represent the thresholds of uncertainty and prediction probability, respectively. When $g_{i}$ is l, it means that sample *i* is filtered as a pseudo-labeled sample.

In order to select as many trainable samples as possible on the premise of ensuring the accuracy of pseudo-labeling, a multi-accumulation strategy is proposed in this paper. The pseudo-label samples selected in the previous round were combined with the training samples to update the AWPN, and the updated AWPN was used for a new round of pseudo-label screening, which was accumulated layer-by-layer until the conditions for stopping were met. If incorrect pseudo-labeling is added to the shallow network, the error information will accumulate in the iteration up to the deep network layer, greatly reducing the accuracy of fault diagnosis. Therefore, a timely stop accumulation strategy is the key to ensure the accuracy of the model. The labeled training set is used to judge whether the network is degraded. If the addition of pseudo-labeling reduces the accuracy of the AWPN on the training set, it indicates that the false pseudo-labeling causes network degradation, and the accumulation strategy is stopped. The accumulation strategy stops in two cases: 1. the filtered pseudo-labeling sample is empty; 2. network degradation.

### 3.4. Overview of the Proposed Method

The semi-supervised few-shot learning based on an adaptive prototypical network and multiple accumulation of pseudo-labeling samples is proposed in this article, with the pseudo code shown in Algorithm 1.


**Algorithm 1:** The proposed learning strategy

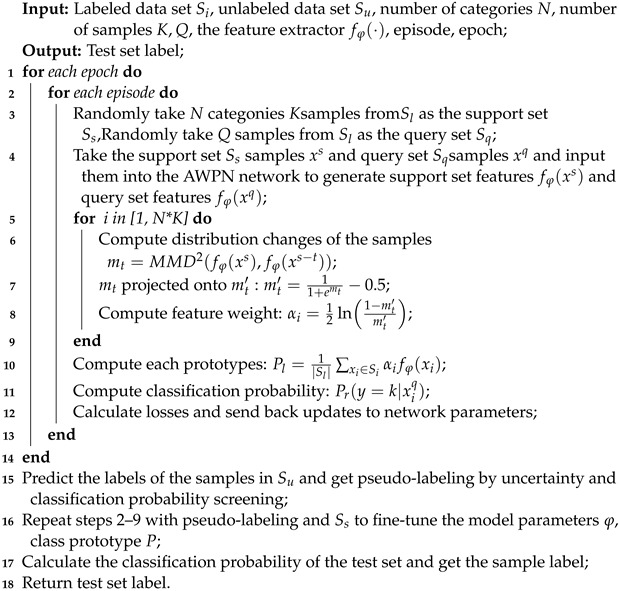




## 4. Results of Experiments

### 4.1. Dataset Description

**Figure 3:**
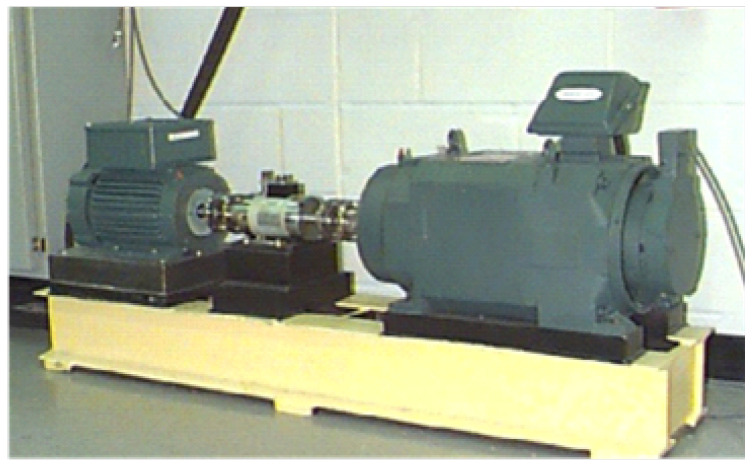
CWRU bearing test bed.

Dataset A: The CWRU dataset is a classic dataset in bearing fault diagnosis and is widely used in the field of bearing fault diagnosis [38]. The fault types are divided into inner race fault (IF), outer race fault (OF), roller fault (RF), and normal state (N). Each type of fault is composed of vibration signals collected under different working conditions, with a sampling frequency of 12 kHz. In this experiment, a sliding window with a length of 2048 and a step size of 80 was used to obtain vibration data samples. The CWRU dataset experimental platform is shown in Figure 3, and the data introduction is shown in Table 1.

**Figure 4:**
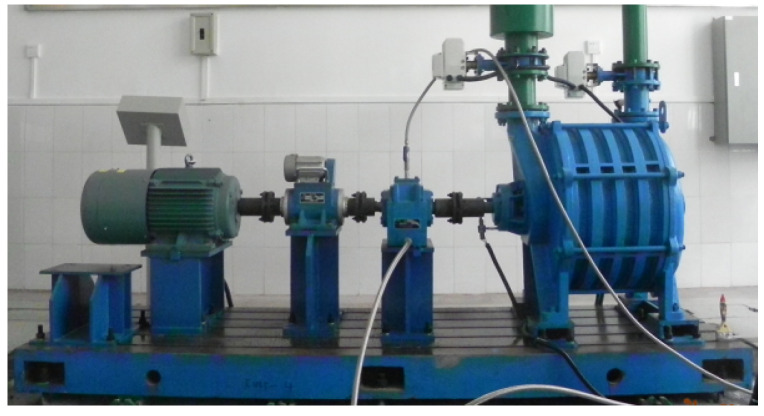
Petrochemical experiment platform.

Dataset B: The petrochemical dataset is collected in industrial environments. Under industrial operating conditions, the collected vibration signals usually contain more noise [39]. For example, environmental noise, temperature changes, equipment aging, and other factors may have an impact on vibration signals, making the data more in line with the actual conditions of industrial operation. The petrochemical experiment platform is shown in Figure 4. The dataset contains six different fault states, normal, defect (s) in gearwheels (F1), defect (s) in gearwheels along with the outer-ring wear of the left-hand side bearing (F2), defect (s) in gearwheels along with the inter-ring wear of the left-hand side bearing (F3), defect (s) in gearwheels along with the absence of balls on the left-hand side bearing (F4), defect (s) in pinions along with the defect (s) in gearwheels (F5). A vibration sensor mounted on the bearing seat is used to collect vibration acceleration signal data, and the data introduction is shown in Table 2.

Dataset C: The IMS bearing dataset was constructed by the Intelligent Maintenance Systems Center at the University of Cincinnati in the United States [40]. The bearings are subjected to simulated degradation tests from a bearing test bench consisting of a 2000 RPM motor and four bearings mounted on the same shaft. During the experiment, the vibration signal generated by the bearing during the operation of the test bench is collected by installing an accelerometer on the bearing seat, with a sampling frequency of 20 kHz. This dataset is the full life cycle data of bearings. In multiple running fault tests, three bearings occurred: outer race failure (ORF), inner race failure (IRF), and ball failure (BF). The data introduction is shown in Table 3.

### 4.2. Results and Analysis

In order to prove the effectiveness of the proposed, three methods are selected for comparative experiments:

The kernel principal analysis-based semi-supervised prototypical network (K-kernel PN) [24], in which the classical prototypical network is used to realize fault diagnosis with the principal component analysis of the Gaussian kernel function to process the original vibration signal.

The robust re-weighting prototypical networks (WProNet) [41], which incorporate a re-weighting mechanism to set a weight for each summation item.

(IPNet) [19], which adjusts the weights of prototypical networks based on the largest average differences between data.

In order to ensure the fairness of comparison, the parameters of the three data sets are the optimal parameters provided in the paper, and all the results are obtained by running 10 times. A time window of length 1024 and step length 128 is used to divide the original oscillation signal. Each type of fault randomly selects 500 samples as the training set, with 1400 samples as the test set. The unlabeled test set is also used for the pseudo-label screening. Table 4, Table 5 and Table 6 show the simulation results of the four methods in three data sets.

It can be seen from the results in Table 4, Table 5 and Table 6 that the proposed method is superior to other models in all tasks of small sample classification. WProNet and IPNet use weighted prototypical networks, but the weighting method is too simple and does not use pseudo-labeling technology to increase the number of available samples, resulting in poor performance in the petrochemical dataset. Although pseudo-labeling technology is used in PSSPN, a single criterion cannot guarantee the accuracy of screening, so the accuracy is lower than that of the proposed method, which proves the effectiveness of the AWPN and dual-threshold multi-accumulation pseudo-labeling screening strategy.

### 4.3. Ablation Experiment

To demonstrate the effectiveness of the adaptive weighting module and pseudo-labeling screen module, we conducted ablation experiments on three datasets, and the experimental results are shown in Table 7. The original prototypical network incorporates pseudo-labeling screening (PNIPS) to compare the effectiveness of the adaptive weighting module. The AWPN does not include a pseudo-labeling module to compare the effectiveness of the pseudo-labeling screen. The experimental results show that the addition of adaptive weights can generate a more representative class prototype. The screen of pseudo-labeling can increase the amount of valid data. Therefore, both can improve the classification accuracy of the mode.

### 4.4. Visualization Analysis

**Figure 5:**
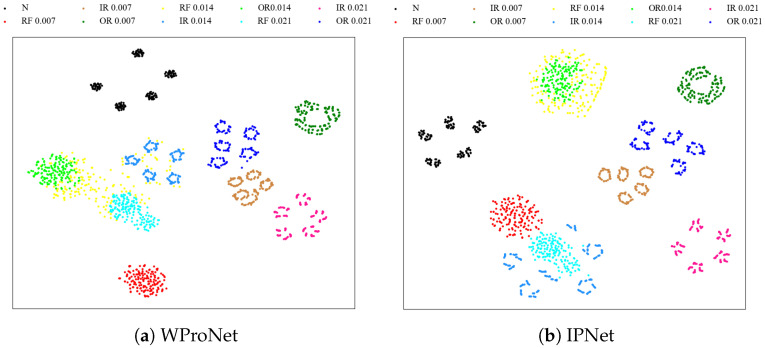

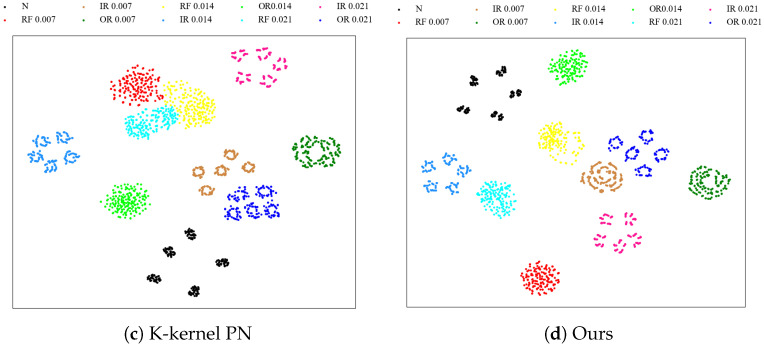
Visualization of CWRU data set features under four models.

The t-distributed random domain embedding (t-SNE) is used to display the visualization images of the CWRU dataset in the 10-way 5-shot scenario under the four models mentioned in Section 4.2. The experimental results are shown in Figure 5; as can be seen from the figure, the same type of features in our model are close to each other, and the overall boundary is relatively obvious. Different class features are far from each other, resulting in the best classification performance. However, the classification performance of other models still needs to be improved, with a few features of different classes crossing each other and a few features overlapping together. Possible reasons may be the misclassification of the model, as well as the prototype generated from the extracted features not maximizing the distance between different classes as much as possible.

**Figure 6:**
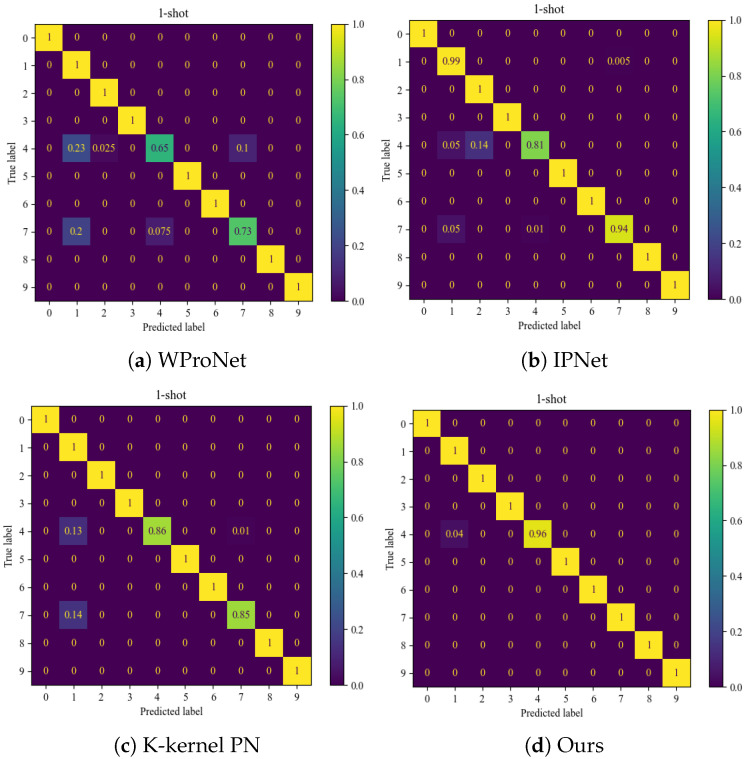
Confusion matrix of CWRU data set under four models.

Figure 6 shows the confusion matrices obtained from the classification of the CWRU dataset in the case of 10-way 5-shot. According to the testing results, the WProNet and K-kernel PN models performed poorly in classifying label 4 (inner race fault with a fault diameter of 0.007) and label 7 (outer race faults with a fault diameter of 0.007), while performing well for other categories. The classification performance of IPNet is better than that of WProNet and K-kernel PN, but it still performs poorly in classifying label 4. WProNet and IPNet do not use pseudo-labeling techniques to increase the number of trainable samples, which can lead to overfitting in the training. K-kernel PN has conducted pseudo-label screening and uses the original prototype network, causing the network performance to degrade. Among these four methods, the proposed method had the best classification effect.

### 4.5. Related Parameters

**Figure 7:**
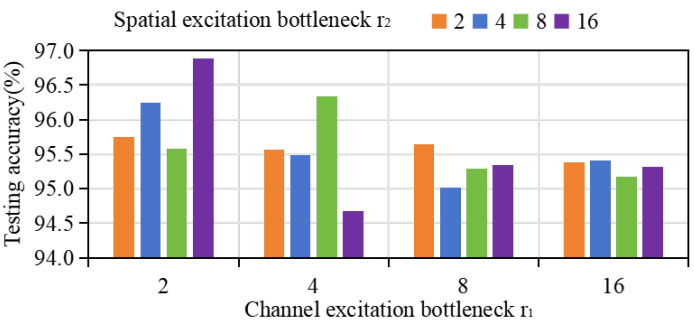
Accuracy of CWRU data set under different values of hyperparameters $r_{1}$ and $r_{2}$.

The hyperparameters involved here are mainly the bottlenecks $r_{1}$ and $r_{2}$ representing channel excitation and spatial excitation, and $\tau_{q}$ and $\tau_{p}$ represent uncertainty and category probability thresholds. Appropriate $r_{1}$ and $r_{2}$ can improve the efficiency of feature extraction, enabling the extracted features to accurately depict different types of fault expressions, and $\tau_{q}$ and $\tau_{p}$ determine the accuracy of pseudo-labeling screening. Therefore, the selection of parameters will greatly affect the performance of the model. For $r_{1}$ and $r_{2}$, the values are usually {2,4,8,16}. In order to remove the influence of parameters in pseudo-labeling screening, the adaptive weighted prototypical network (without pseudo-labeling screening) and CWRU dataset were selected for parameter evaluation, and the results were obtained as shown in Figure 7; the optimal parameters are $r_{1}=2$ and $r_{2}=16$. The detailed system parameter settings in this article are shown in Table 8 and Table 9.

### 4.6. Validity of Dual-Threshold Pseudo-Labeling Screen

**Figure 8:**
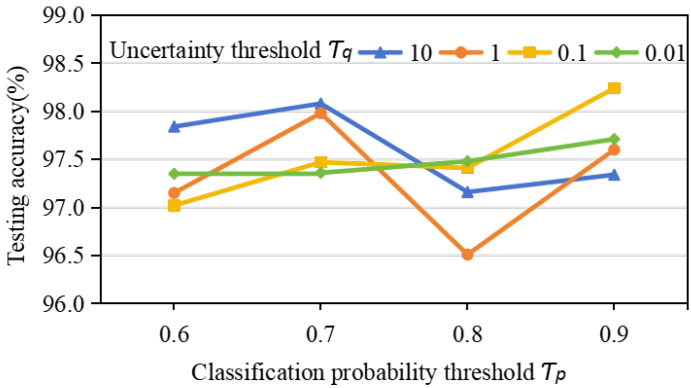
Accuracy of CWRU data set under different values of hyperparameters $\tau_{q}$ and $\tau_{p}$.

The range of uncertainty threshold $\tau_{q}$ is {10,1,0.1,0.01}, and the range of output probability threshold $\tau_{p}$ is {0.6,0.7,0.8,0.9}. The results obtained by adding a pseudo-labeling screen module to the AWPN with determined parameters are shown in Figure 8, and the optimal parameters are $\tau_{q}=0.1$ and $\tau_{p}=0.9.$

### 4.7. Effectiveness of Dual-Threshold Pseudo-Labeling Screen

The output probability is usually selected as the criterion for false label screening, and a single threshold is prone to producing false pseudo-labeling. In this paper, the dual-threshold of output probability and uncertainty are adopted, and multiple selection strategies are adopted to screen out more samples without reducing the accuracy of pseudo-labeling. To demonstrate the effectiveness of the dual threshold strategy, a single output probability threshold and a single uncertainty threshold were selected as the comparison terms for the dual threshold strategy. In the application of the CWRU dataset, the accuracy of the model is shown in Table 10.

## 5. Conclusions

The article proposed a pseudo-labeling multi-screening-based semi-supervised learning method for few-shot fault diagnosis to improve the generalization ability under limited label samples. The proposed method consists of a squeeze and excitation block-based feature extractor, an AdaBoost adaptation-based prototypical clustering, and a pseudo-labeling multi-screening strategy. The feature extractor of the proposed method achieves effective information by squeeze and excitation; high accuracy pseudo-labeling samples are generated with pseudo-labeling screening to expand the number of trainable data points to verify the effectiveness of the proposed method compared with the existing methods in two ways (5-way and 10-way) with three shots (1-shot, 5-shot, and 10-shot). A comparison result of feature visualization and confusion matrix with other related scenarios illustrated that the proposed method has significant performance in the different shots diagnosis tasks. However, our proposed method still has certain limitations, which reduced the time cost in the process of pseudo-labeling multi-screening. Therefore, in future research, it is necessary to study the method with simpler structure and lower complexity.

## Figures and Tables

**Table 1:** Description of CWRU dataset.

Fault Location	Load	Fault Diameter (Inch)	Fault Label
N		0	0
	0	0.007	1
RF		0.014	2
	1	0.021	3
		0.007	4
IF	2	0.014	5
		0.021	6
	3	0.007	7
OF		0.014	8
		0.021	9

**Table 2:** Description of petrochemical dataset.

Fault Location	Normal	F1	F2	F3	F4	F5
Fault label	0	1	2	3	4	5

**Table 3:** Description of IMS dataset.

Fault Location	Normal	ORF	IRF	BF
Fault label	0	1	2	3

**Table 4:** Average accuracies of the results for CWRU (%).

CWRU	5-Way			10-Way		
	1-Shot	5-Shot	10-Shot	1-Shot	5-Shot	10-Shot
WProNet	92.76 ± 0.11	92.14 ± 0.08	95.18 ± 0.06	87.57 ± 0.09	89.84 ± 0.04	90.13 ± 0.03
IPNet	93.42 ± 0.11	95.03 ± 0.06	94.48 ± 0.07	75.96 ± 0.12	91.41 ± 0.04	93.82 ± 0.03
K-kernel PN	92.63 ± 0.12	95.24 ± 0.07	97.81 ± 0.04	89.72 ± 0.38	94.65 ± 0.16	97.05 ± 0.07
Ours	98.94 ± 0.05	99.29 ± 0.02	98.19 ± 0.03	97.40 ± 0.05	98.19 ± 0.02	99.09 ± 0.01

**Table 5:** Average accuracies of the results for petrochemical (%).

CWRU	5-Way			10-Way		
	1-Shot	5-Shot	10-Shot	1-Shot	5-Shot	10-Shot
WProNet	88.86 ± 0.19	89.88 ± 0.14	90.99 ± 0.12	80.74 ± 0.15	83.54 ± 0.06	82.04 ± 0.04
IPNet	88.55 ± 0.19	93.33 ± 0.11	91.14 ± 0.11	80.60 ± 0.13	83.62 ± 0.06	85.04 ± 0.04
K-kernel PN	91.89 ± 0.11	97.07 ± 0.01	98.12 ± 0.08	89.61 ± 0.30	96.23 ± 0.06	97.77 ± 0.08
Ours	92.11 ± 0.16	97.36 ± 0.11	98.46 ± 0.03	91.67 ± 0.13	96.50 ± 0.06	97.72 ± 0.04

**Table 6:** Average accuracies of the results for IMS (%).

CWRU	5-Way			10-Way		
	1-Shot	5-Shot	10-Shot	1-Shot	5-Shot	10-Shot
WProNet	92.97 ± 0.18	99.59 ± 0.02	99.86 ± 0.01	93.35 ± 0.12	99.23 ± 0.02	99.43 ± 0.01
IPNet	96.53 ± 0.13	97.62 ± 0.06	99.83 ± 0.01	95.16 ± 0.10	99.24 ± 0.02	99.35 ± 0.01
K-kernel PN	98.92 ± 0.07	99.56 ± 0.01	99.95 ± 0.00	99.38 ± 0.04	99.79 ± 0.00	97.77 ± 0.01
Ours	99.66 ± 0.04	99.82 ± 0.01	99.97 ± 0.01	99.72 ± 0.02	99.91 ± 0.01	99.98 ± 0.00

**Table 7:** Results of ablation experiment (%).

Method	CWRU	IMS	Petrochemical
	10-Way 5-Shot	4-Way 5-Shot	6-Way 5-Shot
PNIPS	94.14 ± 0.06	98.11 ± 0.01	98.67 ± 0.06
AWPN	96.89 ± 0.03	99.03 ± 0.02	97.98 ± 0.06
Ours	98.19 ± 0.02	99.71 ± 0.01	99.50 ± 0.06

**Table 8:** The feature extractor parameters.

Layer	Parameter	Out Shape
Input		1024 × 1
Conv1D/BN/ReLu/Maxp	channels = 64, c_size = [3], c_str = [1], p_size = [2], p_str = [1]	512 × 64
Conv1D/BN/ReLu/Maxp	channels = 64, c_size = [3], c_str = [1], p_size = [2], p_str = [1]	256 × 64
Conv1D/BN/ReLu/Maxp	channels = 64, c_size = [3], c_str = [1], p_size = [2], p_str = [1]	128 × 64
Conv1D/BN/ReLu/Maxp	channels = 64, c_size = [3], c_str = [1], p_size = [2], p_str = [1]	64 × 64
Conv1D/BN/ReLu/Maxp	channels = 64, c_size = [3], c_str = [1], p_size = [2], p_str = [1]	31 × 64
Dropout/Flatten	Units = 1984, dropout_rate = 0.2	1984

**Table 9:** The scSE parameters.

Operation	Parameter	Out Shape
Channel_input		1984
Expand_dim	dim = −1	1984 × 1
Channel_expansion	channel = 64	1984 × 64
Avgpool	dim = 0	1 × 64
Squeeze	dim = −1	1 × 4
Excitation/ReLu	units = 64 (Dense1)	1 × 64
Fusion/Sigmoid	units = 1984 (Dense2)	1984
Spatial_inputt		1984
Expand_dim	dim = −1	1984 × 1
Channel_expansion	channel = 64	1984 × 64
Conv/Sigmoid	channels = 1, c_size = [1], c_str = [1]	1984 × 1
Squeeze	dim = −1	992 × 4
Excitation/ReLu	units = 1984 (Dense3)	1984 × 1
Fusion/Sigmoid	units = 64 (Dense4)	1984 × 4
Max	(Channel_input, Spatial_input)	1984 × 1
Dim_reduction	Units = −1	1984

**Table 10:** Accuracy of the model under different hyperparameters (%).

	Without $\tau_{p}$	$\tau_{p}=0.6$	$\tau_{p}=0.7$	$\tau_{p}=0.8$	$\tau_{p}=0.9$
Without $\tau_{q}$	None	96.76 ± 0.026	98.03 ± 0.021	97.59 ± 0.023	97.70 ± 0.023
$\tau_{q}=10$	96.84 ± 0.026	97.84 ± 0.022	98.08 ± 0.021	97.16 ± 0.026	97.34 ± 0.024
$\tau_{q}=1$	96.94 ± 0.026	97.15 ± 0.025	97.98 ± 0.021	96.51 ± 0.027	97.60 ± 0.023
$\tau_{q}=0.1$	96.92 ± 0.026	97.02 ± 0.025	97.47 ± 0.024	97.41 ± 0.024	98.24 ± 0.019
$\tau_{q}=0.01$	97.73 ± 0.022	97.35 ± 0.024	97.36 ± 0.024	97.48 ± 0.024	97.71 ± 0.022

## Data Availability

Data are contained within the article.
